# *RUNX* super-enhancer control through the Notch pathway by Epstein-Barr virus transcription factors regulates B cell growth

**DOI:** 10.1093/nar/gkw085

**Published:** 2016-02-15

**Authors:** Andrea Gunnell, Helen M. Webb, C. David Wood, Michael J. McClellan, Billy Wichaidit, Bettina Kempkes, Richard G. Jenner, Cameron Osborne, Paul J. Farrell, Michelle J. West

**Affiliations:** 1 School of Life Sciences, University of Sussex, Falmer, Brighton BN1 9QG, UK; 2 Department of Gene Vectors, Helmholtz Center Munich, German Research Center for Environmental Health, Marchioninistraße 25, 81377 Munich, Germany; 3 German Centre for Infection Research (DZIF), Partner site Munich, Helmholtz Center Munich, German Research Center for Environmental Health, Marchioninistraße 25, 81377 Munich, Germany; 4 University College London Cancer Institute, Paul O'Gorman Building, 72 Huntley Street, London WC1E 6BT, UK; 5 Department of Genetics & Molecular Medicine, King's College London School of Medicine, Guy's Hospital, Great Maze Pond, London SE1 9RT, UK; 6 Department of Medicine, Virology Section, St Mary's Hospital Campus, Imperial College, London W2 1PG, UK

## Abstract

In B cells infected by the cancer-associated Epstein-Barr virus (EBV), *RUNX3* and *RUNX1* transcription is manipulated to control cell growth. The EBV-encoded EBNA2 transcription factor (TF) activates *RUNX3* transcription leading to RUNX3-mediated repression of the *RUNX1* promoter and the relief of RUNX1-directed growth repression. We show that EBNA2 activates *RUNX3* through a specific element within a −97 kb super-enhancer in a manner dependent on the expression of the Notch DNA-binding partner RBP-J. We also reveal that the EBV TFs EBNA3B and EBNA3C contribute to *RUNX3* activation in EBV-infected cells by targeting the same element. Uncovering a counter-regulatory feed-forward step, we demonstrate EBNA2 activation of a *RUNX1* super-enhancer (−139 to −250 kb) that results in low-level *RUNX1* expression in cells refractory to RUNX1-mediated growth inhibition. EBNA2 activation of the *RUNX1* super-enhancer is also dependent on RBP-J. Consistent with the context-dependent roles of EBNA3B and EBNA3C as activators or repressors, we find that these proteins negatively regulate the *RUNX1* super-enhancer, curbing EBNA2 activation. Taken together our results reveal cell-type-specific exploitation of *RUNX* gene super-enhancers by multiple EBV TFs via the Notch pathway to fine tune *RUNX3* and *RUNX1* expression and manipulate B-cell growth.

## INTRODUCTION

The mammalian runt-related family of transcription factors (TF) *(RUNX*) is encoded by three separate genes (*RUNX1, RUNX2* and *RUNX3*) located on different chromosomes that play crucial roles in the control of a range of developmental and differentiation processes (1). *RUNX* genes have distinct patterns of tissue-specific expression, but all bind the same DNA consensus site, through heterodimerization with the non-DNA binding CBFβ protein, to activate or repress transcription (2,3). Disruption or misregulation of *RUNX* expression is associated with a wide range of human tumours (1). *RUNX1* is frequently translocated in myeloid and lymphoid malignancies, with fusion of *RUNX1* to the Ets family *TEL* TF in B-cell acute lymphoblastic leukaemia and to *ETO* in acute myeloid leukaemia (4). *RUNX2* is essential for osteogenesis and linked to osteosarcoma (5) and *RUNX3* is inactivated in a variety of solid tumours (1). *RUNX1* and *RUNX3* play important roles in regulating haematopoesis with loss of *RUNX1* resulting in defective T and B-cell development and embryonic lethality in mice and loss of *RUNX3* resulting in altered T-cell differentiation profiles (1). For all *RUNX* genes transcription initiates from one of two promoters located distal (P1) or proximal (P2) to the translation start site that give rise to protein isoforms that differ in their amino termini and alternative splicing generates further isoforms with functional differences. *RUNX1* transcription is also regulated by a Gata2 and Ets protein-controlled +23 kb intronic enhancer in mouse cells and by an equivalent haemopoietic-cell-specific enhancer (RE1) in human cells (6,7). The 173 kb region between P1 and P2 encompassing RE1 also functions as a CDK7-dependent RUNX1 super-enhancer in T-cell acute lymphoblastic leukaemia cell-lines (8).

Epstein-Barr virus (EBV) is a key driver in the development of a wide range of lymphomas including Burkitt's (BL), Hodgkin's and Diffuse Large B-cell (9). Its ability to immortalize resting B cells *in vitro* reflects its oncogenic properties and results in the generation of permanently proliferating lymphoblastoid cell lines (LCLs) in which the virus persists in its latent form (10). Latently infected LCLs express a limited set of EBV proteins comprising six nuclear antigens (EBNAs 1, 2, 3A, 3B, 3C and leader protein) and three latent membrane proteins (LMP1, 2A and 2B). In addition to regulating viral latent gene transcription, EBNA2 and the EBNA3 family of TFs (3A, 3B and 3C) drive growth transformation through epigenetic reprogramming of the host B cell (11–16). These viral TFs do not bind DNA directly, however, but hijack B cell TFs in order to access viral and cellular gene regulatory elements. The best characterized of these interactions is between EBNA2, 3A, 3B and 3C and the Notch signalling pathway DNA-binding protein RBP-J (CBF1, CSL, Su(H)) (17–21). The interaction between EBNA2, 3A, 3C and RBP-J is essential for EBV-driven B cell growth demonstrating a central role for RBP-J in cellular gene reprogramming (22–24). In reporter assays, EBNA3 proteins inhibit RBP-J dependent gene activation by EBNA2 in manner involving competitive binding to RBP-J (18,21,25), although EBNA2 and EBNA3 proteins appear to bind RBP-J at different sites on the protein (26–28).

EBNA2 and EBNA3C also interact with the cellular TF PU.1 and EBNA2 activation of the EBV LMP1 promoter requires the presence of both PU.1 and RBP-J binding sites, indicating a role for PU.1 in the regulation of at least a subset of genes (29–31). Interestingly, the LMP1 promoter PU.1 site resembles a composite PU.1/IRF element and these composite sites are implicated in the EBV type-specific regulation of specific cellular genes by EBNA2 (16,32). A binding site for EBF1 is also required for activation of the LMP1 promoter by EBNA2 (33).

EBNA2 is best characterized as a transcriptional activator and harbours a classical acidic activation domain (34), although repressed gene targets have been identified (35,36). EBNA3 proteins function as activators and repressors of transcription, curbing EBNA2 activation through their associations with RBP-J, but also regulating transcription through EBNA2-independent mechanisms. Their role in epigenetic silencing through the polycomb repressor complex-mediated H3K27me3 chromatin silencing mark has been well studied (14–15,37). We and others have shown that EBNA2 and EBNA3 proteins predominantly target cellular genes through their associations with long-range regulatory elements (15–16,32–33,38–39). Studying the influence of EBNA binding on long-range enhancer-promoter interactions we demonstrated that EBNA3 proteins can repress cellular gene transcription by preventing enhancer-promoter loop formation (anti-looping) or by a repression mechanism involving the formation of loops between target gene promoters and distal EBNA3-bound sites (16).

Here, we identify the key elements within *RUNX3* and *RUNX1* super-enhancers through which EBNA2 and EBNA3 proteins control *RUNX* expression to manipulate B cell growth. Our data demonstrate that the Notch pathway component RBP-J is required for EBNA2 activation of *RUNX3* and reveal additional coactivation of *RUNX3* by EBNA3B and 3C. We also uncover direct feed-forward control of a novel cell-type specific *RUNX1* super-enhancer region by EBNA2 through RBP-J-dependent mechanisms and show that at *RUNX1*, EBNA3B and 3C attenuate this activation.

## MATERIALS AND METHODS

### Cell lines

All cell lines were routinely passaged twice-weekly and cultured using the conditions previously described for each line. The DG75 cell-line originates from an EBV negative BL (40) and the RBP-J (CBF1) knockout derivative cell-line was described previously (41). The EBV-positive latency III BL cell line Mutu III (clone 48) derives from Mutu I latency I BL cells grown in culture and has been described previously (42). The EBV immortalized LCL GM12878 is an ENCODE Tier 1 cell line obtained from the Coriell Cell Repositories. The PER253 B95.8 LCL was provided by Dr H. Long and has been described previously (43).The EBV negative BL31 BL cell line series infected with wild-type recombinant EBV bacmids or EBNA 3A, 3B and 3C individual or triple knockout and revertant bacmids has been described previously and was kindly provided by Prof M. Allday (13).

### Plasmid construction

The *RUNX3* P2 promoter from −737 to +44 relative to the transcription start site was amplified using the following primers (5′ ACGCCGCGAGGCCTGCAAGAT 3′ and 5′ GGCCGCAGCCCCAGAACAAA 3′) and cloned into PCRII-TOPO. Sequencing detected a single nucleotide change relative to the published sequence (CTTCCGCCCC has become CTTCCACCCC). A Hind III/Xho I *RUNX3* P2 promoter fragment was then cloned into pGL3 basic (Promega) to generate pGL3RUNX3P2. *RUNX3* enhancer regions were amplified from bacmid RP11-349B5 (Bacpac Resources) using primers designed to introduce 5′ NheI and 3′ XhoI sites (Supplementary Table S1). The enhancer 1 (E1) polymerase chain reaction (PCR) product (which also has an Xho1 site at its 5′ end), was digested with XhoI and cloned into the Xho1 site of pGL3Runx3P2 to generate pGL3RUNX3P2 E1. All other enhancer regions (E2, E3, E4, E4 + 5, E5 and E6) were amplified by PCR and products digested with NheI and XhoI. NheI/XhoI enhancer fragments were then cloned into the Nhe1 and Xho1 sites of pGL3Runx3P2 to generate pGL3RUNX3P2E2, pGL3RUNX3P2E3, pGL3RUNX3P2E4, pGL3RUNX3P2E4+5, pGL3RUNX3P2E5 and pGL3RUNX3P2E6. pGL3RUNX3P2E1-6, containing all six enhancer regions, was cloned sequentially as follows: E4 + 5 was excised from pGL3RUNX3P1E4 + 5 as an Nhe1/EcoRV fragment, the 5′ overhang from Nhe1 was filled in using Klenow (New England Biolabs) and the blunt-ended fragment cloned into pGL3Runx3P2E6 digested with EcoRV to generate pGL3Runx3P2E4–6. E3 was excised from pGL3RUNX3P2E3 as an Nhe1/EcoRV fragment and cloned into pGL3RUNX3P2E4-6 digested with EcoRV using the same strategy to give pGL3Runx3P2E3–6. E2 was then excised from pGL3Runx3P1E2 and inserted into EcoRV digested pGL3Runx3P2E3–6 as a blunted Nhe1/EcoRV fragment to give pGL3RUNX3P2E2–6. Finally, E1 was excised from pGL3RUNX3P2E1 as an XhoI fragment, and cloned into pGL3RUNX3P2E2–6 digested with XhoI to generate pGL3RUNX3P2E1–6.

pGL3RUNX1P1 was described previously and contains the *RUNX1* P1 promoter from −151 to +100 (44). Enhancer regions 2–5 were amplified from bacmid RP11-749I9 (Bacpac Resources) using primers designed to introduce 5′ NheI and 3′ XhoI sites (Supplementary Table S1). PCR products for enhancers 2–5 were digested with NheI/XhoI and cloned into pGL3RUNX1P1 cut with Nhe1/Xho1 to give pGL3RUNX1P1E2, pGL3RUNX1P1E3, pGL3RUNX1P1E4 and pGL3RUNX1P1E5. RUNX1 enhancer regions 1 and 6 were synthesized using GeneArt Strings^®^ (Invitrogen). Sequences were derived from ENCODE hg19, chr21:36561619–36562555 and chr21:36669712–36670621 respectively and provided in pMA-T vectors. Enhancer region 1 (E1) was excised from the pMA-T vector using AflIII and the 5′ overhangs filled with Klenow and inserted into pGL3RUNX1P1 digested with SmaI to generate pGL3RUNX1P1E1. Enhancer region 6 (E6) was excised from the pMA-T vector using MluI and SmaI and the 5′ overhang filled with Klenow prior to insertion into the SmaI site of pGL3RUNX1P1 to generate pGL3RUNX1P1E6. pGL3RUNX1P1E1 + 4 + 6 containing RUNX1 enhancers 1, 4 and 6, was cloned sequentially as follows: E1 was excised from pGL3RUNX1P1E1 as an NheI/EcoRV fragment, blunt ended using Klenow and cloned into pGL3RUNX1P1E4 digested with EcoRV to generate pGL3RUNX1p1E4 + 1. E6 was excised from pGL3RUNX1P1E6 as an EcoRV fragment and cloned into pGL3RUNX1p1E4 + 1 digested with EcoRV to generate pGL3RUNX1p1E6 + 4 + 1. pGL3RUNX3P2E3Δ1 was created by digesting pGL3RUNX3P2E2 with BglII/Xho1 and replacing the excised fragment with a GeneArt™ Strings BglII/Xho1 fragment with the 441 bp hg19 chr1:25 348 801–25 349 241 region of enhancer 2 region deleted.

### Site-directed mutagenesis and deletion

The Q5^®^ Site Directed Mutagenesis Kit (New England Biolabs) was used to generate enhancer mutations. All primers were designed using NEBaseChanger™ software (Supplementary Table S1). To create the *RUNX3* enhancer 2 deletion mutant (Δ2) 391 nt from 25349261–25349614 inclusive were deleted by designing primers that PCR out from the edges of this region in pGL3RUNX3E2. The NF-κB motif in *RUNX3* enhancer 2 was mutated by designing primers to make the required substitution (GCAGGGAAGGCCCCA to GCAGGGAAGGGAATA).

### Transient transfections

For *RUNX1* and *RUNX3* promoter reporter assays, DG75 cells were electroporated with plasmid DNA at 230 V and 950 μF (BioRad Gene Pulser II) and luciferase assays carried out as described previously (45) using sequential injection on a Glowmax multi detection system (Promega). Cells were transfected with 2 μg of the pGL3 luciferase reporter plasmids and 0.5 μg pRL-CMV (Promega) as a transfection control, in the absence or presence of 10 or 20 μg of the EBNA2-expressing plasmid pSG5 EBNA2A. One tenth of each transfection was processed for western blotting to analyse protein expression levels.

### Western blotting

Immunoblotting was carried out as described previously (45,46) using the following antibodies: anti-actin 1/5000 (A-2066, Sigma), anti-EBNA2 PE2 (gift from Prof M. Rowe) 1/300, anti-RUNX1 1/40 (PC-285, Calbiochem), anti- RUNX3 1/200 (SC101553, Santa-Cruz) or anti-RBP-J 1/2000 (SC28713X, Santa-Cruz). Western blot quantification was carried out using Li-COR Image studio software, either directly from images captured using the Li-COR Odyssey Imaging system or with JPEG images generated by scanning of autoradiographs. Signals were adjusted for background, and normalized to the signal for actin.

### ChIP-QPCR

ChIP-QPCR was carried out as described previously for EBNA2, EBNA3A, EBNA3B and EBNA3C using antibodies verified as specific for each EBNA (16,43,46) and primer pairs across *RUNX* enhancer regions (Supplementary Table S2) and previously described positive (CTBP2 enhancer) and negative controls (PPIA) (16). RBP-J ChIP was carried out using 4 μl STL84 JK, a rabbit polyclonal antibody to RBP-J (provided by Prof E Kieff), following the protocol previously described for polyclonal antibodies (43) with the exception that protein A Sepharose beads were blocked with 0.5% bovine serum albumin (w/v) in phosphate buffered saline.

### ChIP-sequencing

EBNA2 was immunoprecipitated from 30 × 10^6^ cross-linked GM12878 cells as described previously using the PE2 mouse monoclonal antibody and a rabbit anti-mouse secondary antibody (15,16). A control immunoprecipitation was carried out in parallel using a 1:1 mix of sheep and mouse IgG (Dako). Libraries were prepared using the NEBNext ChIP-seq library prep reagent set for Illumina and NEBNext Index primers (New England Biolabs) and samples subjected to 50 bp single-end read sequencing with an Illumina Genome Analyzer IIx with a total of seven samples per lane. Data analysis was performed as described previously (15,16). Data are available via GEO accession number GSE76869.

### Capture Hi-C

Previously described capture Hi-C data from GM12878 and CD34+ cells was examined for interactions at *RUNX3* that were captured using a 20.1 kb HindIII P1 promoter fragment as bait (chr1:25273787–25293947) (47).

## RESULTS

### EBNA2 binds a functional long-range *RUNX3* super-enhancer

**Figure 1.**
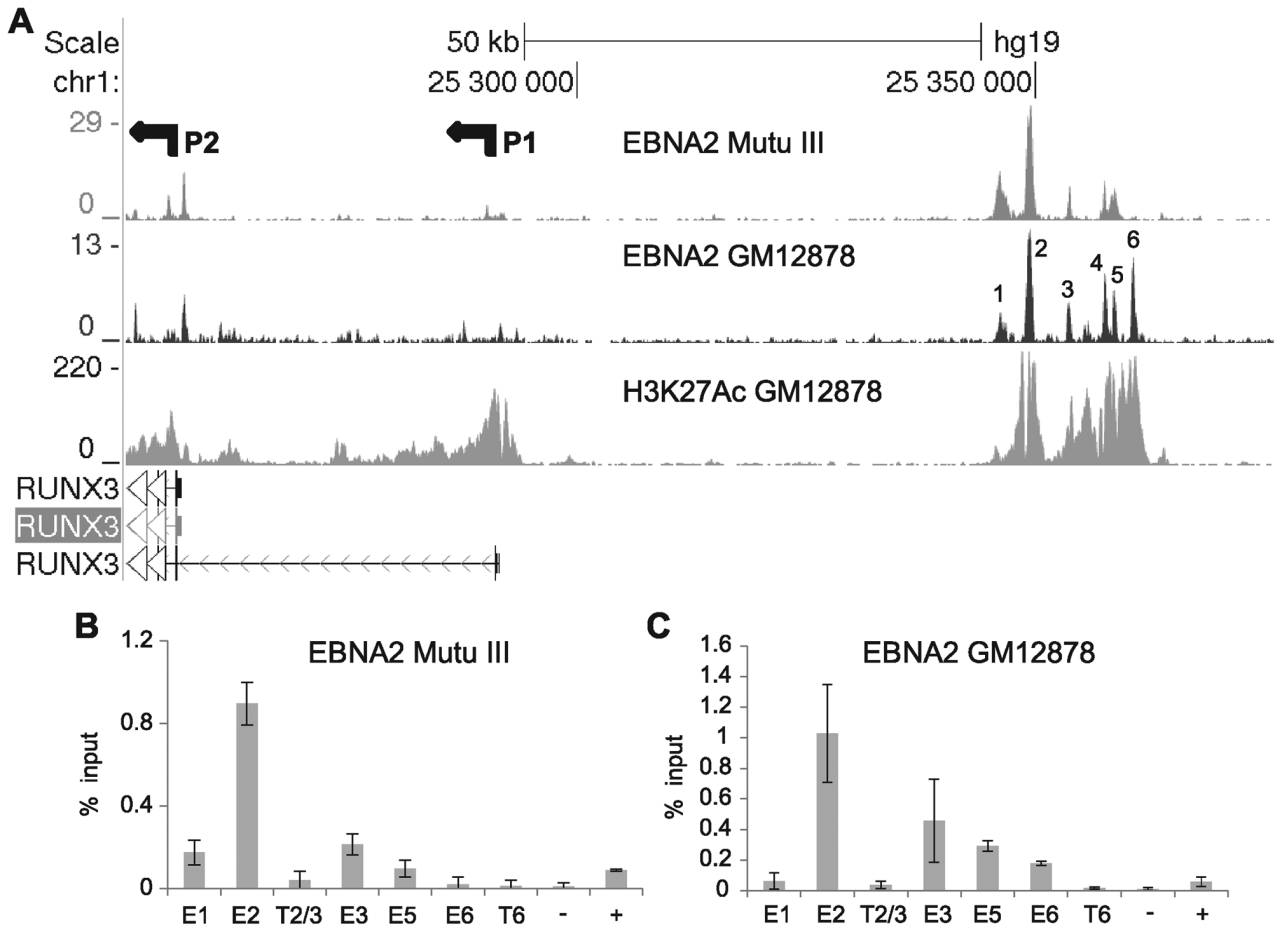
EBNA2 super-enhancer binding at the *RUNX3* locus. (**A**) EBNA 2 ChIP-sequencing reads in Mutu III and GM12878 cells and H3K27Ac signals in GM12878 from ENCODE. The P1 and P2 promoters are indicated; *RUNX3* runs right to left in the human genome. Numbering indicates the major EBNA2 binding sites in the super-enhancer (**B**) ChIP-QPCR analysis of EBNA2 binding in Mutu III cells. Precipitated DNA was analysed using primer sets located at the binding sites (E1, E2, E3, E5, E6) or trough regions between or adjacent to the binding sites (T2/3, T6). EBNA2 binding at the transcription start site of *PPIA* and at the previously characterized *CTBP2* binding site were used as negative (−) and positive binding controls (+), respectively. Mean percentage input signals, after subtraction of no antibody controls, are shown −/+ standard deviation for three independent ChIP experiments. (**C**) ChIP-QPCR analysis of EBNA2 binding in GM12878 cells, as in (B).

To elucidate the mechanism of EBNA2 activation of *RUNX3* in EBV infected cells we examined EBNA2 binding data obtained by ChIP-sequencing from two EBV-infected cell lines, one a Burkitt's lymphoma cell line expressing the full panel of EBV latent genes (Mutu III (16)), and the other the Tier 1 ENCODE EBV-immortalized lymphoblastoid cell line GM12878. We identified a cluster of five to six EBNA2 binding sites in an 18-kb region centred at −97-kb upstream from the *RUNX3* P2 promoter (Figure 1A), the promoter active in EBV-infected B lymphoblastoid cells (44). Five main EBNA2 binding sites were detected in Mutu III cells, but an additional sixth site was present in GM12878 cells. EBNA2 binding at these sites was confirmed by ChIP-QPCR (Figure 1B and C). Examination of ENCODE ChIP-sequencing data for GM12878 revealed high-level H3K27 acetylation (H3K27Ac) across this region (Figure 1A) and chromatin segmentation analysis is consistent with an active regulatory function. Chromatin landscape analysis (dbSUPER, http://bioinfo.au.tsinghua.edu.cn/dbsuper/ (48)) and recent reports classify this region as a highly-active and characteristically large TF binding site cluster indicative of a lineage-specific super-enhancer (49). The super-enhancer classification of this element is thus far restricted to EBV-infected cells (GM12878), early haematopoietic and T cell lineages (CD3, CD56, CD34+ primary, CD8 primary and CD4 T-cell subsets) and not CD19 primary and CD20 B cells, pointing to EBV infection in driving its activation in B cells.

**Figure 2.**
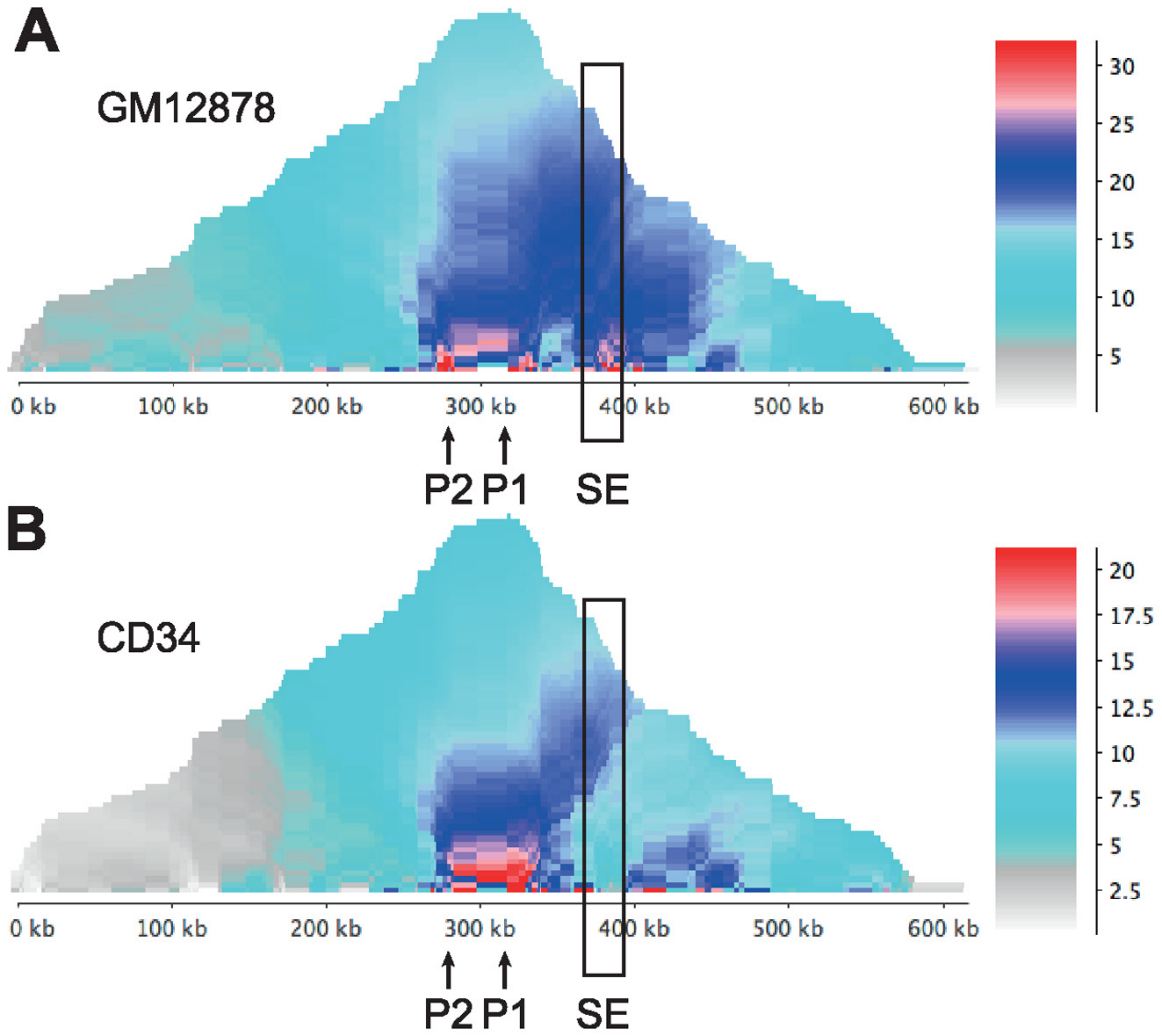
Capture Hi-C interaction analysis at *RUNX3*. (**A**) Domainogram showing the geometric mean of sequencing reads captured by the *RUNX3* P1 promoter bait from a GM12878 Hi-C library. A 600-kb region centred on the P1 promoter is shown. The positions of the P1 and P2 promoters and the super-enhancer region (SE) are indicated. (**B**) Domainogram of *RUNX3* P1 interactions obtained using a CD34+ Hi-C library.

To further examine the functionality of this super-enhancer as a control element for *RUNX3*, we examined long-range promoter interaction data obtained for CD34+ haematopoietic progenitor and GM12878 cells using capture Hi-C (CHi-C). This technique is a newly-developed modification of the Hi-C genome-wide chromosome conformation method that selectively enriches for interactions involving promoters (47). In GM12878 cells, long-range interactions of the *RUNX3* P1 promoter bait captured both the P2 promoter and the super-enhancer region indicating the presence of enhancer-promoter looping consistent with the function of this region as a *RUNX3* enhancer in EBV-infected B cells (Figure 2A). In contrast in CD34+ cells, *RUNX3* promoter-super-enhancer interactions were absent (Figure 2B), indicating that the super-enhancer does not make significant contacts with *RUNX3* promoters in these cells, despite its prediction as a CD34+ cell super-enhancer region.

### The Notch pathway DNA-binding protein RBP-J is necessary but not sufficient for EBNA2 activation of the *RUNX3* super-enhancer

**Figure 3.**
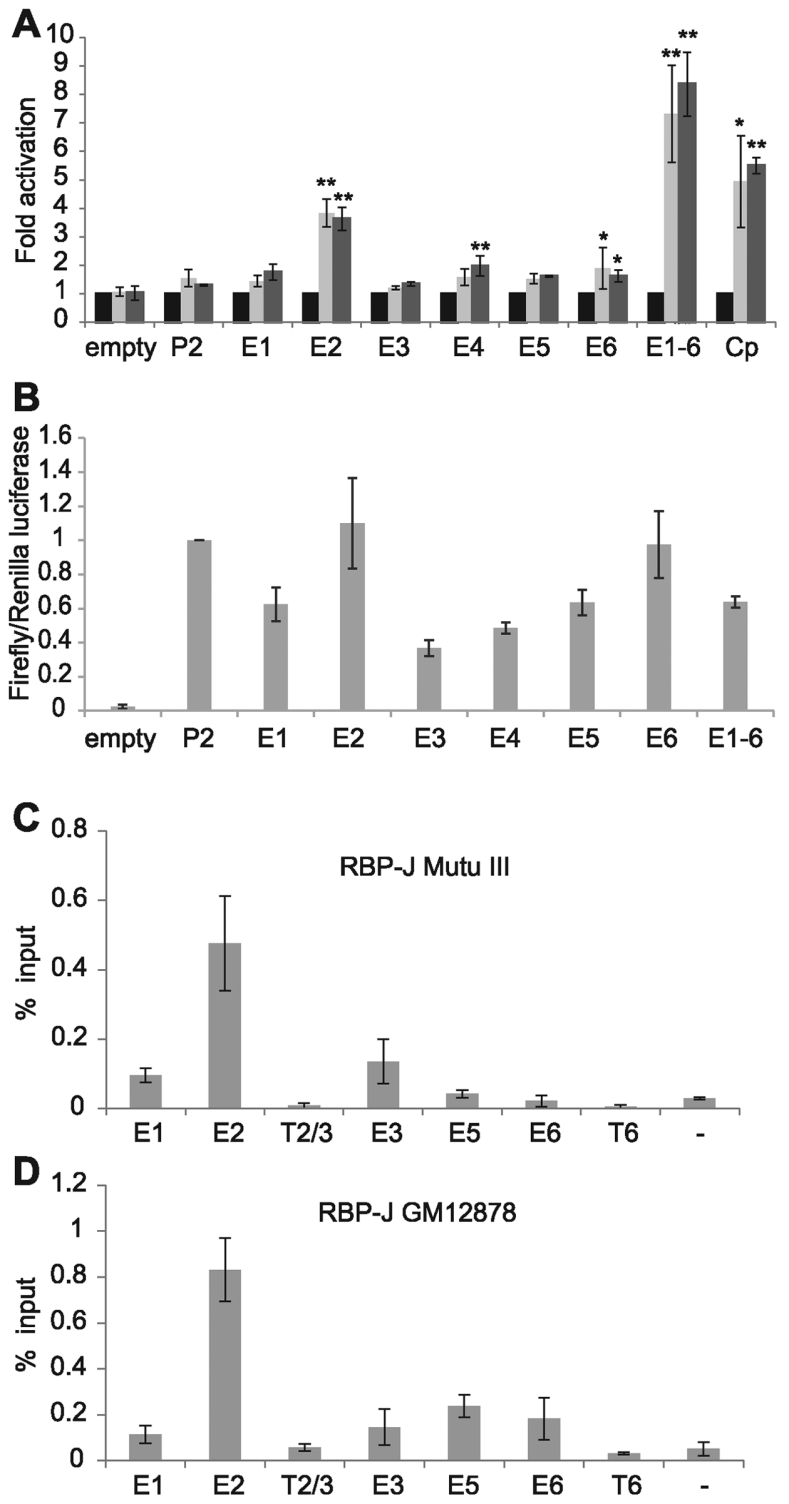
Analysis of *RUNX3* super-enhancer elements. (**A**) Luciferase assay analysis of EBNA2 activation of pGL3basic (empty), the *RUNX3* P2 promoter alone (P2) or P2 in the presence of each enhancer cloned upstream either alone (E1, E2, E3, E4, E5, E6) or in combination (E1–6). EBNA2 activation of the viral C promoter (Cp) was used as a positive control. Cells were transfected with pGL3 reporter constructs and a Renilla luciferase control plasmid in the absence (black bars) or presence of 10 μg (light grey bars) or 20 μg (dark grey bars) EBNA2-expressing plasmid. Results show the mean of three independent experiments +/− standard deviation (**P-value < 0.01 in two-tailed Student's *t*-test, *P-value < 0.05 for fold EBNA2 activation). (**B**) Luciferase assay analysis of basal transcription levels from each reporter construct in the absence of EBNA2. Results show mean Firefly reporter over Renilla control luciferase signals from three independent experiments +/− standard deviation. (**C**) ChIP-QPCR analysis of RBP-J binding in Mutu III cells as in Figure 1. (**D**) ChIP-QPCR analysis of RBP-J binding in GM12878 cells.

To determine which regions of the *RUNX3* super-enhancer mediate EBNA2-responsiveness, we cloned each of the six EBNA2 binding regions into luciferase reporter constructs containing the *RUNX3* P2 promoter and created a single construct containing all six enhancer regions together. Transient transfection of these constructs into B cells in the absence and presence of EBNA2 demonstrated that the 1.5 kb enhancer region 2 was the key mediator of EBNA2 activation (Figure 3A). EBNA2 was able to activate transcription up to 3.8-fold via enhancer 2 and up to 8.4-fold when all enhancer regions were combined (Figure 3A). Enhancers 4 and 6 were activated by EBNA2 up to 2-fold and thus contributed to the increased activation observed in the presence of all six enhancers. The EBV C promoter was used as a positive control and was activated up to 5.5-fold by EBNA2 (Figure 3A). Interestingly, none of the enhancer regions were able to increase basal transcription from P2 in the absence of EBNA2 and some enhancer regions (E1, E3, E4 and E5) decreased basal transcription (Figure 3B). Western blotting confirmed expression of EBNA2 across the different transfections at similar levels (Supplementary Figure S1).

**Figure 4.**
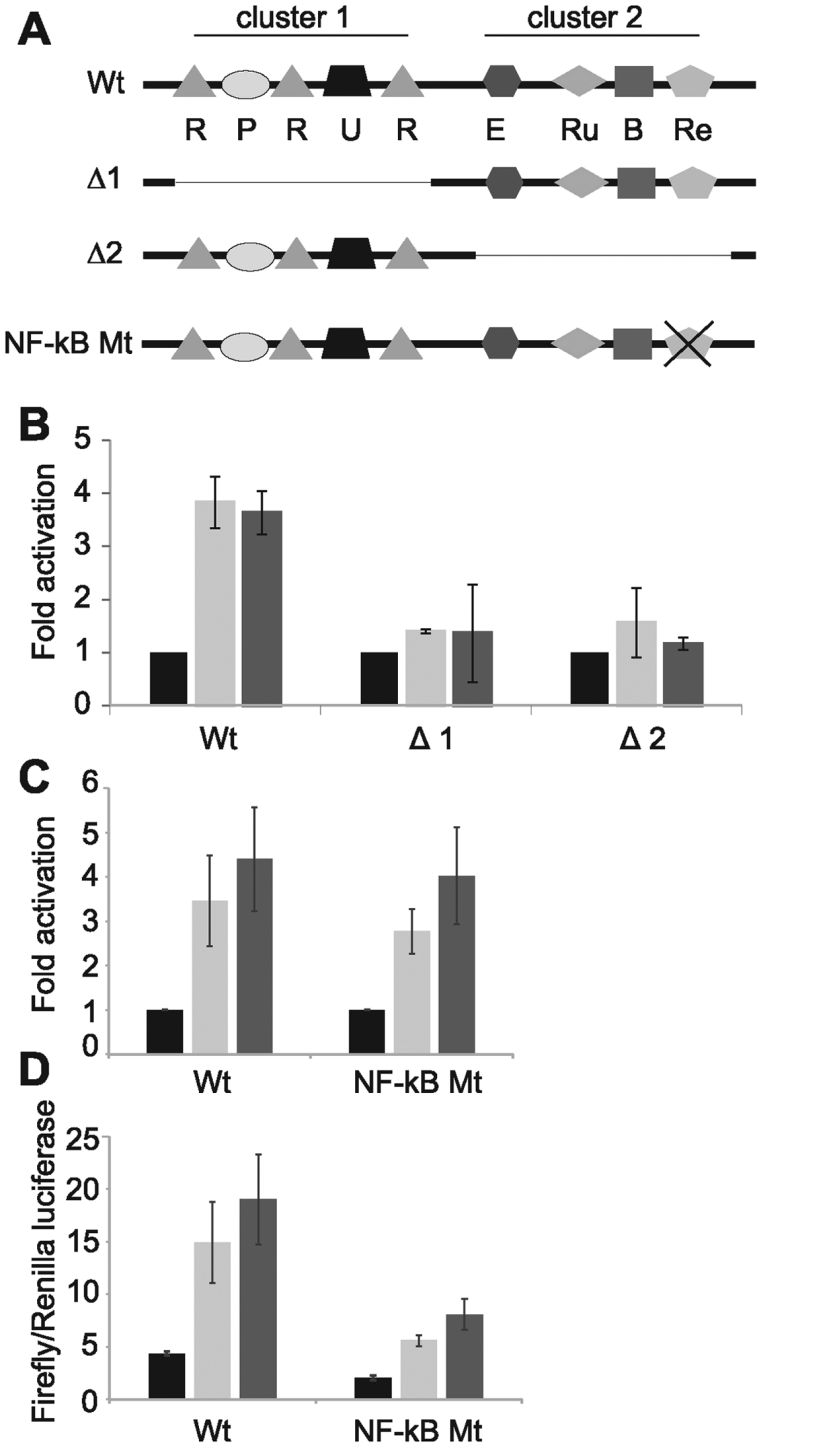
Deletion analysis of *RUNX3* Enhancer 2. (**A**) Diagram of candidate RBP-J sites and TF binding sites identified as bound by each TF via a motif in ENCODE at enhancer 2 (diagram not to scale). R; RBP-J, P; PAX5, U; USF1, E; EBF1, Ru; RUNX3, B; BATF, Re; RelA NF-κB. The regions deleted in the Δ1 and Δ2 mutants are indicated by thin lines. (**B**) Luciferase assay analysis of EBNA2 activation of *RUNX3* P2 plus wild-type enhancer 2 and each enhancer 2 deletion mutant as in Figure 3. Results show mean fold activation from two independent experiments +/− standard deviation in the absence (black bars) or presence of 10 μg (light grey bars) or 20 μg (dark grey bars) EBNA2-expressing plasmid. (**C**) Luciferase assay analysis of EBNA2 activation of *RUNX3* P2 plus wild-type enhancer 2 or enhancer 2 with a mutation in the NF-κB site. Results show mean fold activation from three independent experiments +/− standard deviation as in (A). (**D**) Luciferase assays results from panel C expressed as mean Firefly reporter over Renilla control luciferase signals from three independent experiments +/− standard deviation.

Since enhancer 2 was the major mediator of EBNA2 responsiveness, we next investigated which cellular TFs were responsible for mediating EBNA2 binding and activation via this enhancer. Examination of ENCODE GM12878 Factorbook ChIP-sequencing data for TFs with a motif within binding sites in enhancer 2 revealed EBF1, PAX5, USF1 and RUNX3 had maximum cluster scores for binding, with BATF also bound at high levels (Supplementary Figure S2). A number of other TFs bound at sites with motifs but with lower cluster scores for binding, including the NFκB TF RelA (Supplementary Figure S2) previously implicated as a *RUNX3* transcription regulator (49). Since a previous ChIP-sequencing study detected binding of the cellular Notch pathway DNA-binding protein and EBNA2 binding partner, RBP-J, at the *RUNX3* super-enhancer in the EBV-immortalized LCL IB4 (33), we investigated whether RBP-J bound the *RUNX3* super-enhancer in Mutu III and GM12878 cells. ChIP-QPCR analysis demonstrated that RBP-J bound to the *RUNX3* super-enhancer with maximum binding at enhancer 2 in both cell types (Figure 3C and D) and we identified three candidate RBP-J binding sites within this region (Figure 4A).

To determine which of the candidate cellular TFs may play a role in EBNA2 activation via enhancer 2, we performed an initial deletion analysis. Since the TF binding sites at enhancer 2 were arranged in two clusters, we created enhancer 2 reporter constructs deleted for each cluster (Figure 4A). Transient reporter assays using these deletion mutants demonstrated that loss of either cluster ablated the ability of EBNA2 to activate transcription via enhancer 2 (Figure 4B and Supplementary Figure S1). The *RUNX3* super-enhancer is bound by multiple NF-κB subunits and RUNX3 gene expression is reduced in EBV-infected cells upon inactivation of NF-κB (49), implicating NF-κB in *RUNX3* super-enhancer control. To determine whether the NF-κB site in cluster 2 of *RUNX3* enhancer 2 contributed to EBNA2 activation of the *RUNX3* super-enhancer, we performed luciferase assays using an enhancer 2 reporter construct with the NF-κB site mutated (Figure 4A). We found that the ability of EBNA2 to activate *RUNX*3 P2 transcription via enhancer 2 was unaffected by mutation of this site, indicating that NF-κB subunits do not play a role in mediating the effects of EBNA2 (Figure 4C). Interestingly however, basal transcription levels of the *RUNX3* P2 enhancer 2 construct in the absence of EBNA2 were reduced by ∼50% leading to an equivalent reduction in level of *RUNX3* transcription even in the presence of EBNA2 (Figure 4D). These data therefore support the previous observations that loss of NF-κB leads to a 50% reduction in overall *RUNX3* transcription in EBV-infected cells but rule out a role for NF-κB in *RUNX3* activation by EBNA2 via enhancer 2.

**Figure 5.**
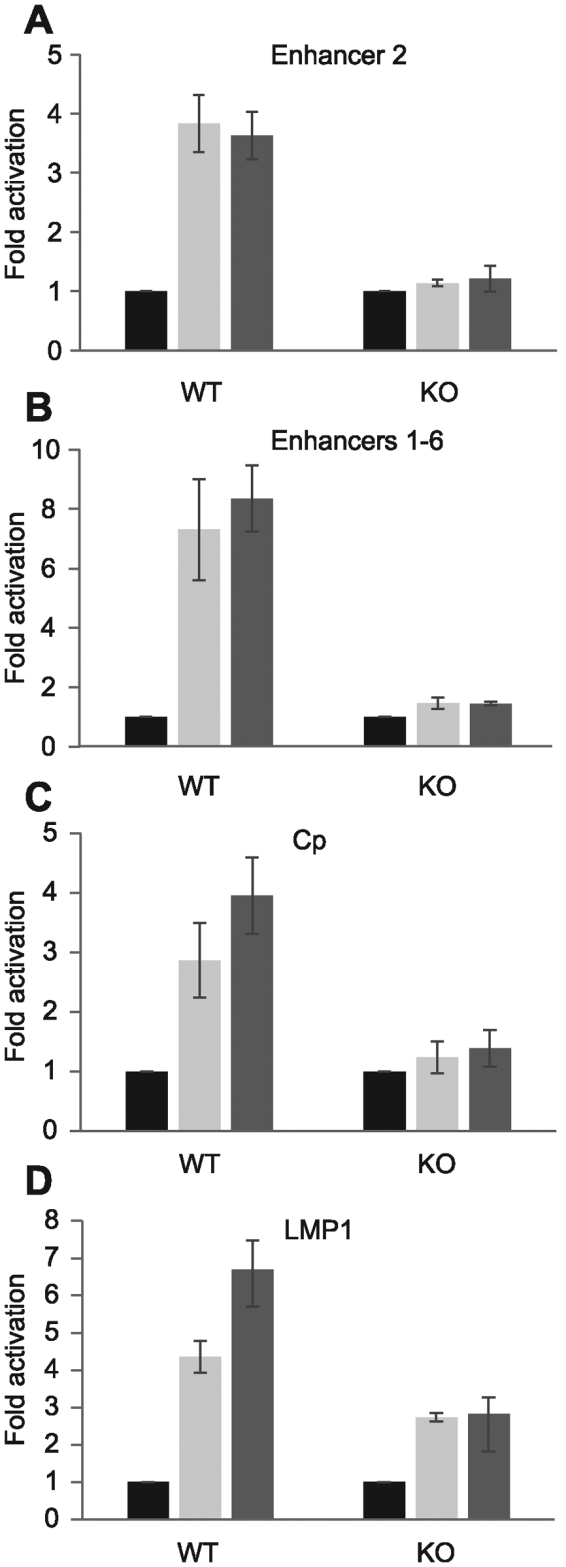
Super-enhancer activation by EBNA2 in DG75 RBP-J knockout cells. Cells were transfected with pGL3 reporter constructs and a Renilla luciferase control plasmid in the absence (black bars) or presence of 10 μg (light grey bars) or 20 μg (dark grey bars) EBNA2-expressing plasmid. Results show the mean of two independent experiments +/− standard deviation (**P-value < 0.01 in two-tailed Student's *t*-test, *P-value < 0.05). (**A**) Luciferase assay analysis of EBNA2 activation of the *RUNX3* P2 promoter in the presence of enhancer 2 (E2) in wild-type DG75 (WT) or RBP-J knockout (KO) cells. (**B**) EBNA2 activation of *RUNX3* P2 promoter plus enhancers 1–6 (E1–6). (**C**) EBNA2 activation of the viral C promoter (Cp) (**D**) EBNA2 activation of the viral LMP1 promoter.

To further investigate a role for RBP-J in the activation of *RUNX3* by EBNA2, we examined the ability of EBNA2 to activate transcription via *RUNX3* super-enhancer elements in an RBP-J knock-out B-cell-line (41). Our results demonstrated that in the absence of RBP-J EBNA2 was no longer able to activate *RUNX3* either via enhancer 2 or all six combined enhancer regions (Figure 5A and B). Control experiments using the EBV C promoter which is activated by EBNA2 in an RBP-J-dependent manner showed the same ablation of EBNA2 activation in RBP-J knockout cells (50) (Figure 5C and Supplementary Figure S1). Consistent with the previously observed partial dependency of the viral LMP1 promoter on RBP-J (30), this promoter was still partially responsive to EBNA2 in RBP-J KO cells, displaying a 2.8-fold response to EBNA2 compared to a 6.7-fold activation in wild-type cells (Figure 5D and Supplementary Figure S1). Taken together, our data indicate that RBP-J is necessary but not sufficient for EBNA2 activation of *RUNX3* and implicate further TFs other than NF-κB in cluster 2 of enhancer 2 in mediating EBNA2 responsiveness in cooperation with RBP-J.

### EBNA3B and EBNA3C coactivate RUNX3 by targeting super-enhancer element 2

**Figure 6.**
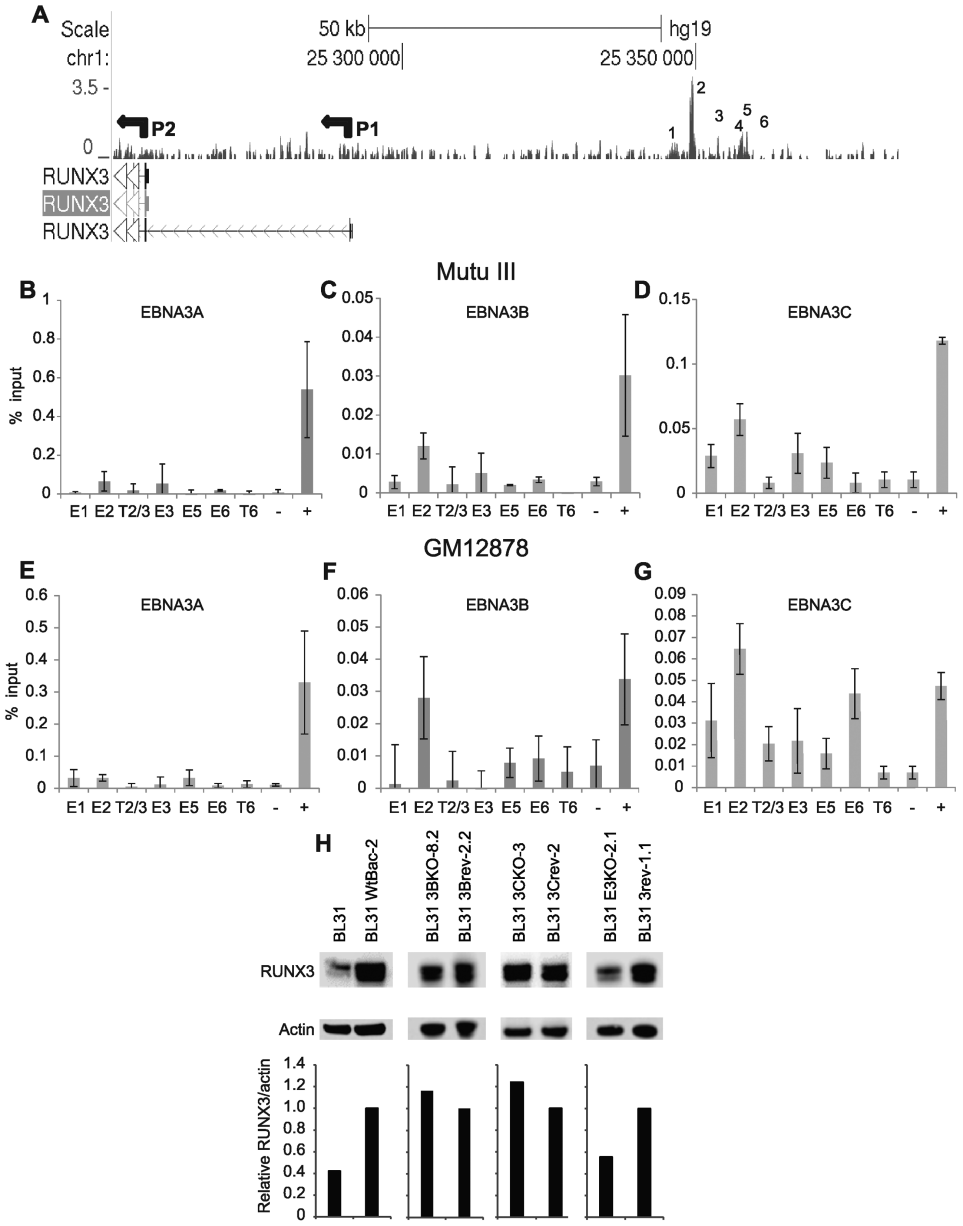
EBNA3A, 3B and 3C super-enhancer binding at the *RUNX3* locus. (**A**) EBNA3 ChIP-sequencing reads in Mutu III cells. The P1 and P2 promoters are indicated. Numbering indicates the major EBNA2 binding sites at the super-enhancer in Mutu III cells. ChIP-QPCR analysis of EBNA3A binding in Mutu III cells (**B**) and GM12878 cells (**E**). ChIP-QPCR analysis of EBNA3B binding in Mutu III cells (**C**) and GM12878 cells (**F**). ChIP-QPCR analysis of EBNA3C binding in Mutu III cells (**D**) and GM12878 cells (**G**). Precipitated DNA was analysed as described in Figure 1. Mean percentage input signals, after subtraction of no antibody controls, are shown −/+ standard deviation for two (EBNA3B in Mutu III) or three independent ChIP experiments. (**H**). Western blot analysis of RUNX3 and actin (loading control) protein expression in uninfected BL31 cells or cells infected with EBNA3B (3BKO), EBNA3C (3CKO) or EBNA3A, 3B and 3C (E3KO) knockout viruses or revertants (rev). *RUNX3* protein levels were quantitated, normalized to actin protein levels and expressed relative to the signal in BL31 cells infected with wild-type recombinant EBV (WtBac-2) or the corresponding revertant viruses.

In addition to the identification of EBNA2 human genome binding sites, our previous ChIP-sequencing experiments also examined binding of EBNA3A, 3B and 3C across the genome (15,16). Examination of EBNA3 binding at the *RUNX3* locus revealed that EBNA3 proteins also target the *RUNX3* super-enhancer. Consistent with the key role of enhancer 2, EBNA3 binding was predominantly localized to enhancer 2 (Figure 6A). Since we have previously demonstrated that distinct subsets of EBNA3 proteins target specific gene regulatory elements, we performed ChIP-QPCR using individual antibodies specific to EBNA3A, 3B and 3C to examine the binding of these three viral TFs at *RUNX3* enhancer 2. We found that only EBNA3B and 3C bound to this region (Figure 6B–G). Examination of previously published microarray analysis (11) confirmed a role of EBNA3 proteins in the regulation of *RUNX3* transcription. Previous analysis of EBV negative BL cells infected with wild type EBV bacmids or EBV bacmids deleted for the EBNA3A, 3B or 3C genes individually or in combination showed that infection with wild-type EBV led to a 3.3-fold upregulation of *RUNX3* mRNA levels, but infection with viruses lacking all EBNA3 proteins resulted in 2.4-fold lower expression than in wild-type EBV infected cells (11). We confirmed that these effects were also evident at the protein level (Figure 6H). *RUNX3* protein levels were upregulated 2.5-fold on infection of EBV negative BL31 cells with wild-type EBV, with cells infected with revertant viruses expressing similar levels to wild type infected cells, as expected (Figure 6H). In contrast, cells infected with a virus lacking EBNA3A, 3B and 3C displayed a 1.8-fold reduction in *RUNX3* protein expression compared to the corresponding revertant cell-line (Figure 6H). Loss of EBNA3B or EBNA3C individually had no effect on the upregulation of *RUNX3* indicating that EBNA3B and EBNA3C can independently activate *RUNX3* transcription at the mRNA or protein level (11) (Figure 6H). *RUNX3* transcription in EBV-infected cells is therefore upregulated through targeting of the upstream super-enhancer by EBNA2, EBNA3B and EBNA3C.

### Feed-forward activation of *RUNX1* by EBNA2 via an upstream super-enhancer

**Figure 7.**
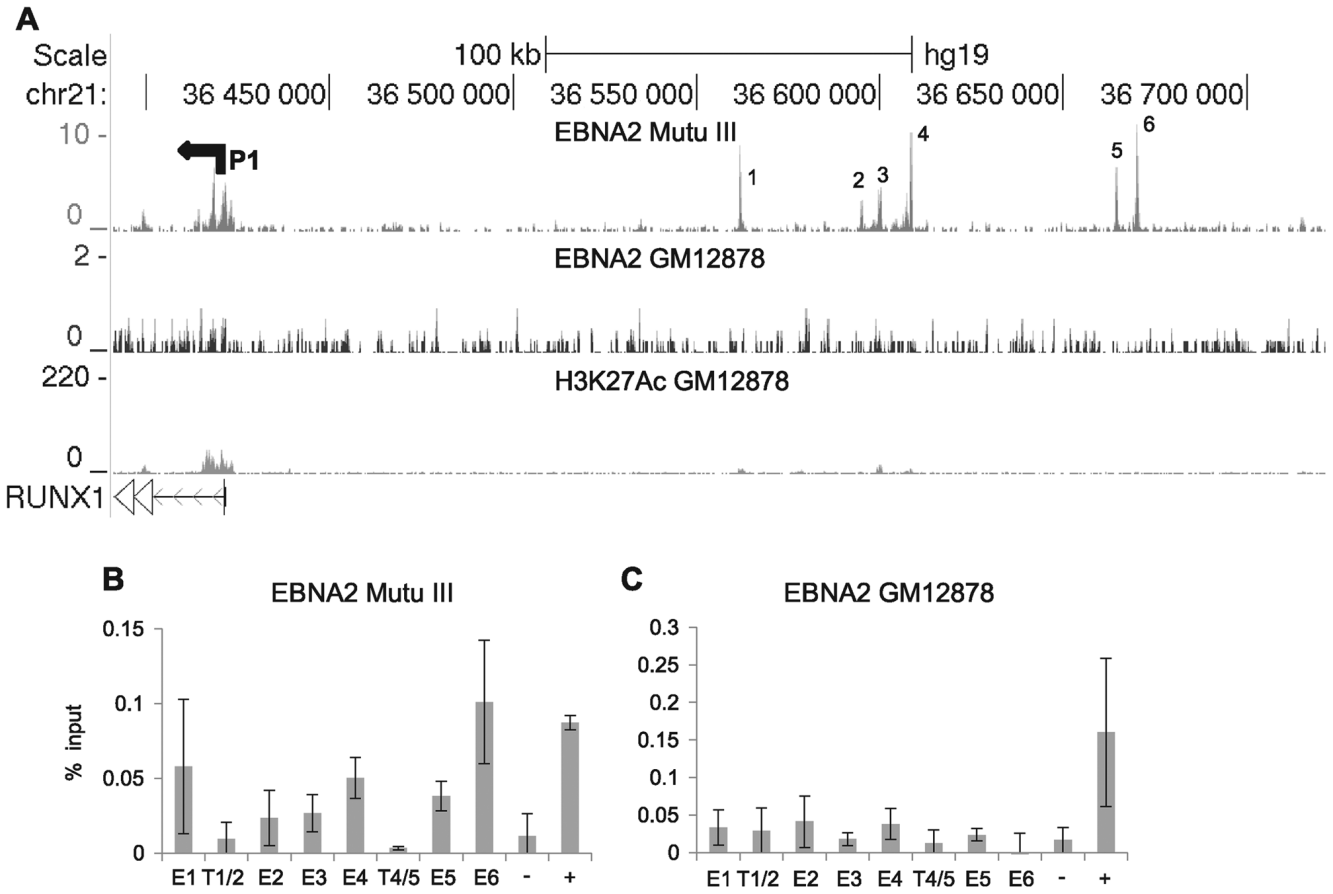
EBNA2 super-enhancer binding at the *RUNX1* locus. (**A**) EBNA 2 ChIP-sequencing reads in Mutu III and GM12878 cells and H3K27Ac signals in GM12878 from ENCODE. The P1 promoter is indicated (P2 is located further downstream and is not shown). *RUNX1* runs right to left in the human genome. Numbering indicates the major EBNA2 binding sites in the super-enhancer (**B**) ChIP-QPCR analysis of EBNA2 binding in Mutu III cells. Precipitated DNA was analysed as in Figure 1 but using primer sets located at the binding sites (E1, E2, E3, E4, E5, E6) or trough regions between the binding sites (T1/2, T4/5). (**C**) ChIP-QPCR analysis of EBNA2 binding in GM12878 cells.

Previous studies have shown that upregulation of *RUNX3* by EBNA2 results in reduced *RUNX1* transcription through the repressive effects of RUNX3 binding to the *RUNX1* P1 promoter, the *RUNX1* promoter active in B cells (44). In addition to this indirect control of *RUNX1* transcription, our ChIP-sequencing analysis has now revealed that EBNA2 also binds to six sites in a region 139–250-kb upstream from *RUNX1* P1 pointing to a role in the direct control of *RUNX1* (Figure 7). Interestingly, EBNA2 appears to target this region in a cell-type specific manner, with binding detected in Mutu III BL cells and not in GM12878 or other LCLs (Figure 7 and data not shown) as confirmed by ChIP-QPCR. This region has not been previously described as a control region for *RUNX1* but analysis revealed that areas within this region encompassing EBNA2 binding sites are classified as super-enhancers in specific cell backgrounds, including the Diffuse large B cell lymphoma cell-lines Toledo and HBL1, the Breast cancer line HCC1954, mammary epithelial cells and skeletal muscle (dbSUPER, http://bioinfo.au.tsinghua.edu.cn/dbsuper/ (48)). Consistent with the lack of H3K27Ac and EBNA2 binding in the GM12878 LCL (Figure 7), this region is not predicted to be a super-enhancer in these cells.

**Figure 8.**
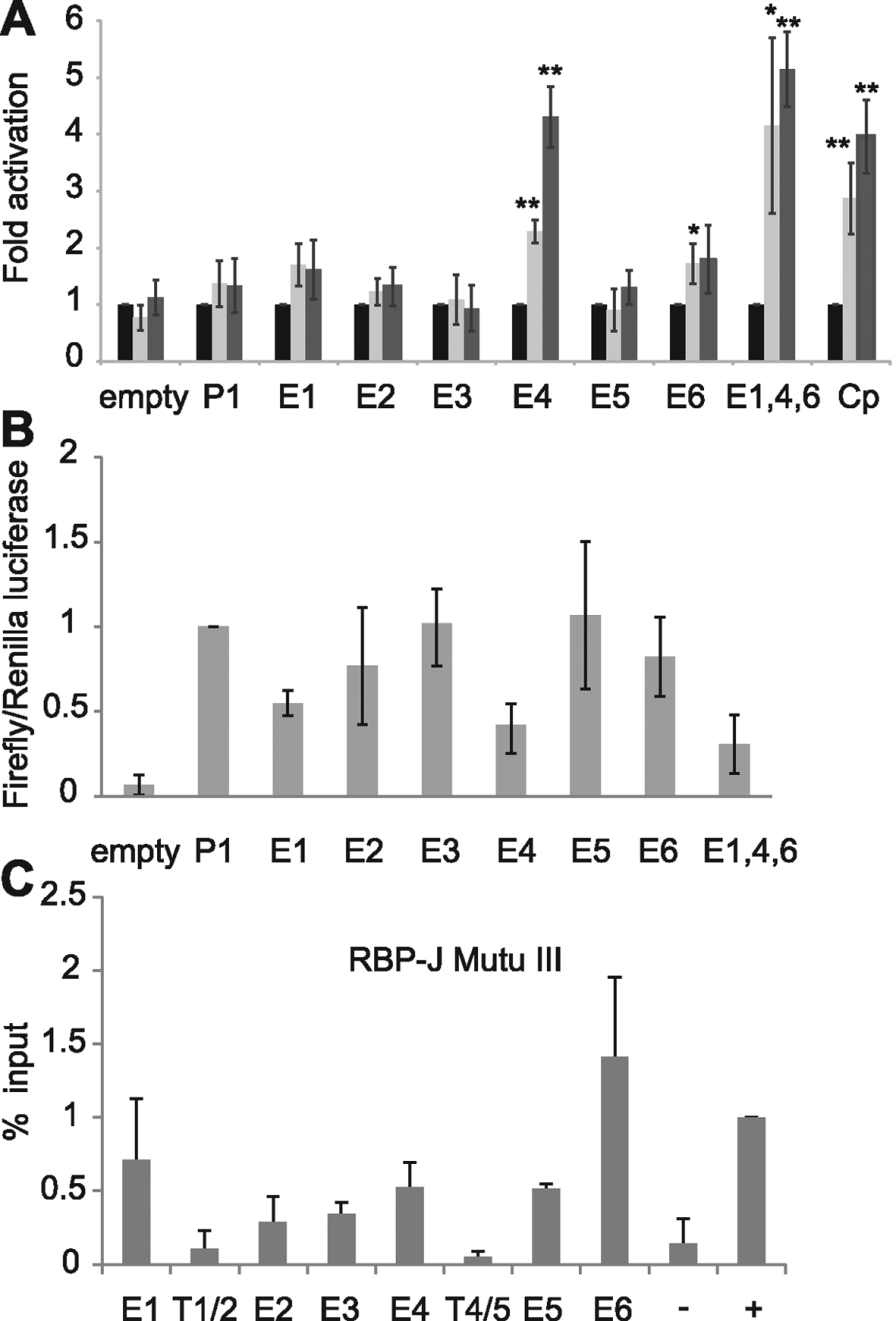
Analysis of *RUNX1* super-enhancer elements. (**A**) Luciferase assay analysis of EBNA2 activation of pGL3basic (empty), the *RUNX1* P1 promoter alone (P1) or P1 in the presence of each enhancer cloned upstream either alone (E1, E2, E3, E4, E5, E6) or with E1, E4 and E6 in combination (E1,4,6). Cells were transfected with pGL3 reporter constructs as in Figure 3. (**B**) Luciferase assay analysis of basal transcription levels from each reporter construct in the absence of EBNA2 as in Figure 3. (**C**) ChIP-QPCR analysis of RBP-J binding in Mutu III cells as in Figure 7.

Cloning the six EBNA2 binding regions into luciferase reporter constructs containing the *RUNX1* P1 promoter revealed that the 2.6-kb Enhancer 4 region was the major determinant of EBNA2 responsiveness in the *RUNX1* super-enhancer, with EBNA2 able to activate transcription via enhancer 4 up to 4.3-fold (Figure 8A). Since enhancers 1 and 6 directed small responses to EBNA2 (although not reaching significance for enhancer 1), we also created a reporter construct containing enhancers 1, 4 and 6 together. EBNA2 activation of this construct was slightly increased to 5-fold (Figure 8A). None of the enhancer regions were able to increase basal transcription from *RUNX1* P1 in the absence of EBNA2 and in fact E1, E4 and E1,4,6 regions decreased basal transcription from this promoter (Figure 8B). Western blotting confirmed expression of EBNA2 across the different transfections at similar levels (Supplementary Figure S1). Our data therefore implicate *RUNX1* super-enhancer targeting by EBNA2 in the activation of *RUNX1* expression in certain cell backgrounds.

### EBNA2 activation of the *RUNX1* super-enhancer requires RBP-J

**Figure 9.**
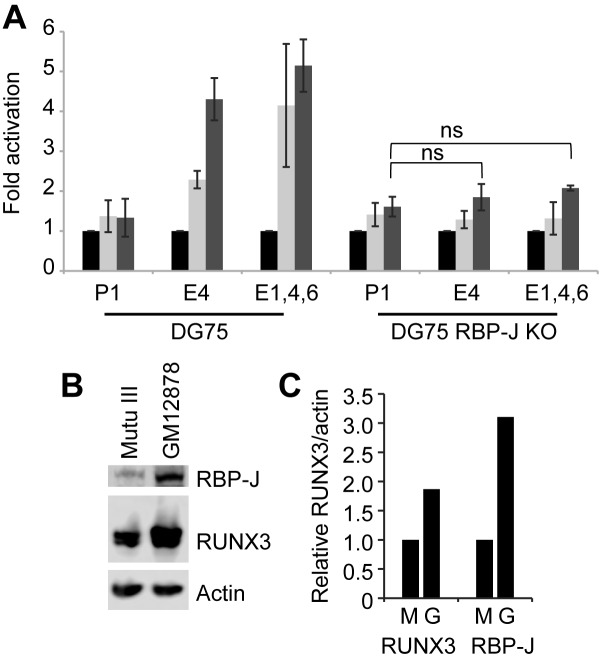
The role of cellular TFs in *RUNX1* regulation. (**A**) Super-enhancer activation by EBNA2 in DG75 RBP-J knockout cells. Activation of the *RUNX1* P1 promoter in the absence or presence of super-enhancer regions E4 or E1,4,6 by EBNA2 in wild-type DG75 cells (three experiments) and DG75 RBP-J knockout cells (two experiments) as described in Figure 5. (**B**) Western blot analysis of RBP-J and *RUNX3* protein expression in Mutu III and GM12878 cells. Actin was used as a loading control. (**C**) RBP-J and *RUNX3* protein levels were quantitated and normalized to actin protein levels. GM12878 (G) levels were expressed relative to the levels in Mutu III cells (M).

To investigate the role of RBP-J in activation of the *RUNX1* super-enhancer region, we examined RBP-J binding at the six *RUNX1* super-enhancer peaks in Mutu III cells using ChIP-QPCR and found that RBP-J bound at the highest levels at enhancer 1, 4 and 6 (Figure 8C). To determine whether EBNA2 was able to activate the *RUNX1* enhancer in the RBP-J knockout B-cell-line, we carried out luciferase assays using a *RUNX1* P1 reporter construct and constructs also containing enhancer 4 or enhancers 1, 4 and 6 (Figure 9A). Similar to our observations for EBNA2 activation of *RUNX3* enhancer elements, we found that EBNA2 activation of *RUNX1* super-enhancer elements was also dependent on RBP-J (Figure 9A). EBNA2 activation via enhancer 4 and enhancers 1, 4 and 6 in RBP-J knockout cells was reduced to levels that were not significantly different from the low-level EBNA2 activation observed for constructs containing the *RUNX1* P1 promoter alone (Figure 9A). To determine whether the lack of EBNA2 binding to *RUNX1* super-enhancer elements in GM12878 cells was due to low RBP-J levels, we examined RBP-J protein expression in Mutu III and GM12878 cells (Figure 9B and C). In fact, RBP-J was expressed at 1.9-fold higher levels in GM12878 so the availability of RBP-J was not a likely explanation. However, *RUNX3* protein levels were also 3-fold higher in GM12878 cells (Figure 9B and C). It is therefore possible that higher levels of RUNX3 lead to increased repression of the *RUNX1* locus in GM12878 cells through the previously described inhibitory effects of RUNX3 on the *RUNX1* P1 promoter (44). Taken together our results indicate that the major mediator of EBNA2 activation of *RUNX1* enhancer regions is RBP-J, pointing to RBP-J as the key cellular TF hijacked by EBV to control *RUNX1* and *RUNX3* transcription in EBV-infected cells.

### *RUNX1* super-enhancer binding by EBNA3B and EBNA3C attenuates EBNA2 activation in BL cells

**Figure 10.**
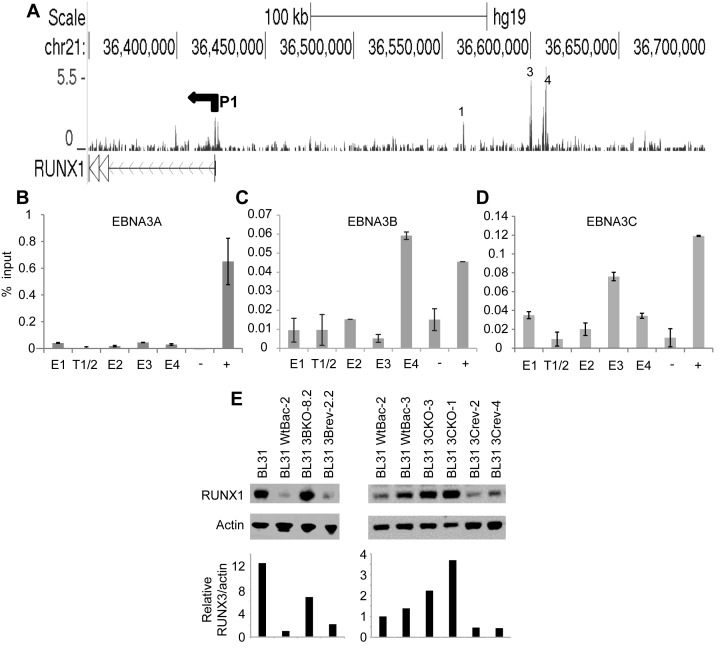
EBNA3A, 3B and 3C super-enhancer binding at the *RUNX1* locus. (**A**) EBNA 3 ChIP-sequencing reads in Mutu III cells. The P1 promoter is indicated. Numbering refers to the location of the EBNA2 binding sites at the super-enhancer in Mutu III cells. ChIP-QPCR analysis of EBNA3A binding (**B**) EBNA3B binding (**C**) and EBNA3C binding (**D**) in Mutu III cells as described in Figure 6 and 1. Mean percentage input signals, after subtraction of no antibody controls, are shown −/+ standard deviation for two independent ChIP experiments. (**E**) Western blot analysis of RUNX1 and actin (loading control) protein expression in uninfected BL31 cells or cells infected with EBNA3B (3BKO), EBNA3C (3CKO) knockout viruses or revertants (rev). *RUNX1* protein levels were quantitated, normalized to actin protein levels and expressed relative to the signal in BL31 cells infected with wild-type recombinant EBV (WtBac-2).

EBNA3 ChIP-sequencing also identified binding of EBNA3 proteins at enhancers 1, 3 and 4 in the *RUNX1* super-enhancer in Mutu III BL cells (Figure 10A). ChIP-QPCR with individual EBNA3 antibodies demonstrated that EBNA3B and EBNA3C but not EBNA3A bound these super-enhancer sites (Figure 10B–D). In fact, different patterns of binding were observed for EBNA3B and 3C, with peak binding of EBNA3B at enhancer 4 and peak binding of EBNA3C at enhancer 3. Our results therefore demonstrate that EBNA2, EBNA3B and EBNA3C target both *RUNX3* and *RUNX1* super-enhancers, but at *RUNX1* binding is cell-type specific. To investigate what effects EBNA3B and 3C binding may have on *RUNX1* expression in BL cells, we again examined microarray data from the EBV negative BL cell series infected with wild-type EBV or EBNA3 knockout viruses (11). These data revealed a clear role for EBNA3B and 3C in repressing *RUNX1*, since in the absence of EBNA3B there was a 2.2-fold increase in *RUNX1* mRNA levels compared to wild-type EBV-infected cells and in the absence of EBNA 3C a 1.8-fold increase in mRNA levels was detected (11). We confirmed that the repressive effects of EBNA3B and EBNA3C on *RUNX1* in this cell series were also evident at the protein level (Figure 10E). Infection of EBV negative BL31 cells with wild-type EBV or revertant viruses reduced *RUNX1* protein expression as expected, but infection with EBNA3B knockout viruses resulted in 6.7-fold higher protein levels than wild-type EBV infected cells (Figure 10E). *RUNX1* protein expression in cells infected with EBNA3C knockout viruses was also 2.2–3.7-fold higher than in cells infected with wild-type EBV (Figure 10E). Since *RUNX3* represses *RUNX1* expression, the role of EBNA3 proteins as activators of *RUNX3* expression would be predicted to lead to a reduction in *RUNX1* mRNA levels. However, in cell lines infected with individual EBNA3B or 3C knockout viruses, no reduction in *RUNX3* mRNA levels were observed (Figure 6H). Thus indirect elevation of *RUNX1* expression due to reduced *RUNX3* expression in these cell lines can be ruled out. The observed effects of EBNA3B and 3C on *RUNX1* expression are therefore most likely to reflect direct effects via their binding to *RUNX1* super-enhancer elements. Our data therefore highlight EBNA3B and 3C as additional regulators of *RUNX3* and *RUNX1* expression in EBV-infected cells. Consistent with their previously reported dual roles as activators and repressors of cellular gene expression (11), in the context of *RUNX* gene regulation they function as activators of *RUNX3* but repressors of *RUNX1*.

## DISCUSSION

**Figure 11.**
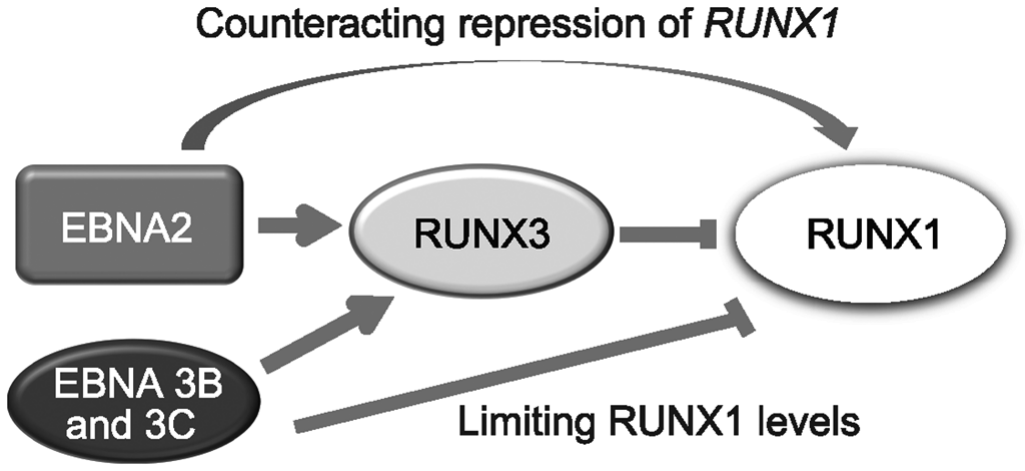
*RUNX* gene regulation by the EBNAs in EBV-infected cells. EBNA2, EBNA3B and EBNA3C activate *RUNX3* expression by binding to a distal upstream super-enhancer. RUNX3 then represses *RUNX1* transcription by binding to the *RUNX1* P1 promoter. EBNA2 can activate *RUNX1* transcription via an upstream super-enhancer, but this is cell-type specific. EBNA3B and EBNA3C repress *RUNX1* transcription in a cell-type specific manner. Total *RUNX1* expression levels depend on the balance between the level of *RUNX3* expression (driving *RUNX1* repression) and EBNA2 activation and EBNA3B and 3C repression mediated via super-enhancer binding.

Studying how *RUNX* transcription is regulated by the EBNA2 and EBNA3 family of EBV TFs in EBV-infected B cells, we have discovered that the Notch pathway DNA-binding protein RBP-J is the key cellular factor hijacked by EBNA2 to direct activation of both *RUNX3* and *RUNX1* transcription via upstream super-enhancers. We also show that EBNA3B and 3C regulate both *RUNX3* and *RUNX1* transcription in opposing directions to fine-tune *RUNX* gene expression in EBV-infected cells, maintaining high *RUNX3* expression and low *RUNX1* expression to prevent inhibition of EBV-immortalized cell growth by RUNX1 (Figure 11).

Analysis of genome-wide sequencing data for long-range chromatin interactions captured by promoter baits (CHi-C) in the GM12878 EBV-immortalized LCL provided clear evidence that the *RUNX3* super-enhancer region interacts with *RUNX3* promoters. This supports the functional relevance of the *RUNX3* super-enhancer as a long-range regulatory element hijacked by EBV to control *RUNX3* transcription. It was not possible to obtain information on *RUNX1* P1 or P2 promoter interactions from the CHi-C GM12878 data set since a far upstream alternative and inactive *RUNX1* promoter was used as bait. Given that the EBNAs do not bind the upstream *RUNX1* super-enhancer region in GM12878 cells, these data would have been likely to provide evidence for a lack of enhancer-promoter interactions in this cell line in any case.

We have delineated the regions of *RUNX* super-enhancers that are the major determinants of EBNA2 responsiveness. For both genes one key region (enhancer region 2 for *RUNX3* and enhancer region 4 for *RUNX1*), corresponding to an EBNA2 binding peak detected in ChIP-sequencing, directs the majority of the EBNA2 activation in enhancer-promoter reporter assays using endogenous *RUNX* promoters. Consistent with the RBP-J-dependent EBNA2 activation of these enhancer elements, they were both bound by RBP-J in EBV-infected cells, along with a number of other B cell TFs, supporting their roles as active regulatory elements. Interestingly, our ChIP-sequencing analysis detected five binding sites for EBNA2 at the RUNX3 super-enhancer in the latency III BL cell line Mutu III, but six sites in GM12878, cells consistent with the histone acetylation pattern at this region in GM12878. Sequencing of these regions from both cell-lines detected a substitution in the Mutu III sequence within a putative low affinity RBP-J motif that reduced RBP-J binding to this site *in vitro* (data not shown). RBP-J bound this site in GM12878 and not Mutu III cells, but at lower levels than enhancer 2, consistent with the weak responsiveness of enhancer region 6 to EBNA2 in reporter assays. It is therefore likely that the lack of binding of EBNA2 to this region in Mutu III cells results from this sequence variation, although the overall activity of the *RUNX3* super-enhancer is unlikely to be particularly affected as a result.

For *RUNX3*, additional cellular TFs in a region adjacent to a cluster of three potential RBP-J sites in enhancer region 2 also appear to play a cooperative role in gene activation by EBNA2 as deletion of this region also attenuates EBNA2 effects. We focused on TFs with ChIP-sequencing data available through ENCODE for the GM12878 LCL that also have a motif at their binding site to reduce the number of candidates. This highlighted EBF1, RUNX3, BATF and RelA as potential cooperative factors. Since NF-κB subunits including RelA have been previously implicated in *RUNX3* transcription control in EBV-infected cells (49), our initial analysis examined the effect of mutating the NF-κB site in enhancer 2. Consistent with this previous study, we found that loss of this site reduced overall enhancer-mediated transcription from the *RUNX3* P2 promoter, but did not impair the ability of EBNA2 to activate transcription. NF-κB binding to enhancer 2 within the *RUNX3* super-enhancer therefore appears to play a key role in maintaining enhancer-driven but EBNA2-independent *RUNX3* transcription in B cells. A full understanding of which regions or TF binding sites within the region adjacent to the cluster of RBP-J sites contribute to EBNA2 activation will require further extensive and systematic mutation of the TF binding sites we have identified based on initial screens for high-level binders with motifs from ENCODE, but likely also deletions and combinations of site mutations across this ∼400-bp region, since many other TFs also bind at lower levels (Supplementary Figure S2), and an even larger number bind in the absence of their canonical motifs.

We have uncovered a new role for EBNA3B and 3C acting alongside EBNA2 in EBV-infected cells to maintain high *RUNX3* expression. The effects of EBNA3 proteins on *RUNX3* transcription would not have been apparent in previous studies that detected *RUNX3* as a direct target for activation by EBNA2 using EBV infected cell-lines expressing conditionally active EBNA2 in the context of the full complement of latent proteins including the EBNA3s, since the loss of EBNA2 alone is clearly sufficient to reduce *RUNX3* transcription (51). The independent ability of EBNA2 to activate *RUNX3* transcription is also evident from the *RUNX3* enhancer reporter assays described here. In contrast to our detection of the binding of endogenous EBNA3B and 3C and not EBNA3A at the *RUNX3* super-enhancer, a previous study documented EBNA3A (and EBNA3C) binding to this region in LCLs infected with recombinant viruses expressing HA/Flag-tagged EBNA3A or 3C proteins (49). There is however, no evidence for a role for EBNA3A in the regulation of *RUNX3* gene expression in BL cells or LCLs to date (11,52).

Our EBNA3B and 3C binding data indicate that the most responsive EBNA2 enhancer, enhancer 2, is the major EBNA3 binding site. Since this enhancer is bound by RBP-J, its activation by EBNA2 is RBP-J dependent, and EBNA3B and 3C bind RBP-J, it is likely that the activation of *RUNX3* transcription by these EBNA3 proteins is also mediated by RBP-J. EBNA2 and EBNA3 proteins associate with RBP-J in a mutually exclusive manner (25) and we have previously shown using re-ChIP that EBNA2 and 3 proteins do not co-occupy the same sites on DNA at the same time (16). In addition, further studies have now convincingly demonstrated that EBNA2 and EBNA3 proteins bind competitively to at least a specific subset of RBP-J-bound cell chromatin sites (53,54). For example, at an intergenic RBP-J-bound enhancer site located between *CXCL9* and *CXCL10* also bound by both EBNA2 and EBNA3A, conditional inactivation of EBNA3A led to a 6-fold increase in EBNA2 binding (53). Recent similar studies of RBP-J-bound genomic sites also bound by either EBNA3A (near *HDAC7* or *CDH1*) or EBNA3C (near *BACH2, JAK1, CXCR5*) found that conditional inactivation of these EBNA3 proteins allowed EBNA2 binding via RBP-J (54). Thus at the *RUNX3* superenhancer, EBNA2 and EBNA3 proteins likely bind this site in different cells in the population used for ChIP-sequencing. For *RUNX3* it appears that EBNA3 binding, like EBNA2 binding, leads to *RUNX3* activation. Consistent with our previous unpublished observations and those of others, we have been unable to reproduce EBNA3 protein-mediated cellular gene activation in reporter assays to date so the mechanisms and factors involved in EBNA3-mediated *RUNX3* activation will require further analysis in a chromatin context that recapitulates the observed *in vivo* effects.

Our ChIP-sequencing analysis uncovered further levels of regulation of the *RUNX* gene network that involve the direct effects of both EBNA2 and EBNA3B and 3C on *RUNX1* transcription, with the effects of EBNA2 again mediated through RBP-J. This direct regulation was not evident from previous studies using transcriptomics approaches alone due to the upregulation of *RUNX3* in EBV-infected cells and the resulting repression of *RUNX1* by *RUNX3* that would have masked any effects of the EBNAs on *RUNX1*. Our data reveal that this direct regulation of the *RUNX1* super-enhancer also occurs in a cell-type dependent manner, consistent with the previously reported cell-type specific nature of super-enhancers (55).

This cell-type specificity could be controlled by the overall level of RUNX3 in the cells which would determine the level of repression of the *RUNX1* locus. Although RUNX3 represses *RUNX1* transcription through a site in the P1 promoter (44), it is possible that this repression would lead to an inhibition of enhancer-promoter interactions and the subsequent ‘closure’ of enhancer chromatin. This would result in reduced binding of cellular TFs like RBP-J and therefore prevent EBNA binding. This theory is supported by the higher level of *RUNX3* expression in GM12878 compared to Mutu III. Further understanding of how RUNX3 may repress the entire *RUNX1* locus and limit super-enhancer activity will require studies of the effects of RUNX3 on *RUNX1* promoter-super-enhancer interactions and cellular TF binding in B cell systems lacking EBNA2 to allow *RUNX3* repression to be examined independently of EBNA2 activation. It is also possible that *MYC* may play a role in the differential activity of the *RUNX1* superenhancer in BL cells compared to LCLs, since BL cells express high levels of *MYC* as a result of the *MYC-IG* translocation. ENCODE ChIP-seq data indicates that both MYC and its partner MAX bind at many sites across the large *RUNX1* super-enhancer region in a number of cell-types. Interestingly, MYC/MAX binding is not detected in this region in the GM12878 LCL where MYC expression is low. Given the co-operative pioneer activity of MYC (56), binding of MYC/MAX to the *RUNX1* super-enhancer region in BLs could increase accessibility for other TFs.

The role of EBNA3B and 3C as repressors of *RUNX1* transcription probably serves as a mechanism to curb high-level activation of *RUNX1* by EBNA2, although why the remaining expression of *RUNX1* induced by EBNA2 does not result in the growth arrest of BL cells is unclear. It is possible that BL cells tolerate higher level *RUNX1* expression than LCLs because BL cell growth is driven by deregulated *MYC*. Since *RUNX1* has conflicting roles in the regulation of cell growth in different backgrounds, it is also possible that there may be some advantage to higher level of expression of *RUNX1* in BL. A full understanding of the role of RUNX1 in controlling B cell growth will await the outcome of genome-wide binding studies that are currently underway.

RBP-J plays an essential role in the immortalization of B cells by EBV and EBNA2, 3A or 3C proteins unable to interact with RBP-J fail to immortalize B cells and/or support the proliferation of infected cells (22–24,57). Our data indicate that regulation of the *RUNX* gene network through super-enhancer control via RBP-J may contribute to the dependency on RBP-J for growth deregulation by EBNA2 and EBNA3C, given the repressive effects of RUNX1 on B cell growth. EBNA3B plays a non-critical role in EBV-mediated transformation since EBNA3B deleted viruses are able to transform B cells (58), although it can also associate with RBP-J (21). Nonetheless EBNA3B is a major controller of cellular gene expression, often acting together with other EBNA3 proteins and may have a tumour suppressive role (11,59). Interestingly, extensive interplay between Notch and RUNX pathways has been reported across a number of developmental systems. Notch signalling during *Drosophila* haematopoiesis induces Lozenge (*RUNX1*) expression in specific precursor cells (60), Notch1 upregulates *RUNX1* transcription in NIH3T3 cells and Notch1-RBP-J null mice display reduced *RUNX1* expression in para-aortic splanchnopleural cells and impaired haemopoietic potential (61). Notch signalling also controls *RUNX1* expression and haematopoietic stem cell development in Zebrafish (62) and *RUNX1* was identified as a direct target induced by activated Notch1 signalling in murine mesodermal cells, but not embryonic stem cells (63). *RUNX3* has also been shown to be a direct target of Notch in murine endothelial cells (64). Our data therefore identify an important role for RBP-J and therefore the Notch pathway in the control of *RUNX* transcription via long-range regulatory elements in human B-cells that is likely to extend to other cell-types.

In summary, our data reveal a complex cross-regulatory *RUNX* gene network controlled by the Notch signalling component RBP-J that is hijacked through interactions with EBV-encoded TFs to fine-tune *RUNX3* and *RUNX1* gene expression and control B-cell growth. We identify *RUNX1* super-enhancer control regions involved in *RUNX1* regulation in B cells that may also function as key control regions in other cell-types. A greater understanding of how *RUNX* genes are controlled by long-range enhancers will help to delineate how these genes are regulated during normal developmental processes and become deregulated in disease.

## Supplementary Material

Supplementary Data

SUPPLEMENTARY DATA
