# Supplementary material for: RUNX super-enhancer control through the Notch pathway by Epstein-Barr virus transcription factors regulates B cell growth
Source: Nucleic Acids Res. 2016 Feb 15;44(10):4636–50. doi: 10.1093/nar/gkw085 (PMC4889917; doi:10.1093/nar/gkw085)
Supplement: Supplementary Data [file gkw085_Supplementary_Data.zip › nar-03672-v-2015-File013.pdf]

Table S1

Amplification of RUNX enhancer regions and deletion and site-directed mutants. Forward (F) and reverse (R) primers

Plasmid	Primers
pGL3RUNX3P2E1	**F** 5′-TAGCACTCGAGCAGTAATGACCCTGCCGAAG<br>**R** 5′-TAGCAGCTAGCAGGGCTTCCAGTGAGAGACA
pGL3RUNX3P2E2	**F** 5′-TAGCACTCGAGCATCCTGAACTGCACTGCTC<br>**R** 5′-TAGCAGCTAGCGCATCACATGGCCTAGGTTT
pGL3RUNX3P2E3	**F** 5′-TAGCACTCGAGGGTCCTGCAGAAGCAGAAGT<br>**R** 5′-TAGCAGCTAGCGGGCCACACAGCAAGTAAGT
pGL3RUNX3P2E4	**F** 5′-TAGCACTCGAGCTCAGGCTGGAAGTTGGCTA<br>**R** 5′-TAGCAGCTAGCGTCTCATCGTCTCTGCACCA
pGL3RUNX3P2E5	**F** 5′-TAGCACTCGAGGGTGCAGAGACGATGAGACA<br>**R** 5′-TAGCAGCTAGCCTCAGATGGCTTCACAAGCA
pGL3RUNX3P2E6	**F** 5′-TAGCACTCGAGGAGAAGCTGGTCCCATCACT<br>**R** 5′-TAGCAGCTAGCGAGATTTGCCACCTCTCACC
pGL3RUNX1P1E2	**F** 5′-GCCGCTAGCGCAGGTGATTCAGCAGCTCTCAG<br>**R** 5′-GCCCTCGAGTGGAGGGTAAGCCTGAAAGGGAT
pGL3RUNX1P1E3	**F** 5′-GCCGCTAGCTGATCTCAGCTCACCACAACCTC<br>**R** 5′-GCCCTCGAGTGTATGTTGGGCTTGCTGTATGC
pGL3RUNX1P1E4	**F** 5′-GCCGCTAGCGCTGAGGCAGGAGAATTGCTTGA<br>**R** 5′-GCCCTCGAGATGAGGGACTGTGCATTTAGCCC
pGL3RUNX1P1E5	**F** 5′-GCCGCTAGCGTTCAATAGACGTCGGTGTACTG<br>**R** 5′-GCCCTCGAGGCTGAATTTAGGGCTAAGGCAGT
pGL3RUNX3P2E2Δ2	**F** 5′-ACGGTGGGCCAGGGCACA<br>**R** 5′-AGTGCCCAGGGCAGCCCTTTTG
pGL3RUNX3P2E2 NF-κB	**F** 5′-GGGAATATCGGGCCAGGTTCTCAGG<br>**R** 5′-TTCCCTGCTGGGACTCCTGGGAGGCC

Table S2. ChIP qPCR primers. Forward (F) and reverse (R) primers.

Gene enhancer region	Primers
RUNX3 E1	**F** 5′-CAGCACAGGCCAAGAGAAC<br>**R** 5′-GGTTGTGCCTGACATTGGTA
RUNX3 E2	**F** 5′-TGTCTCCTGCTGTCTGCCTA<br>**R** 5′-TGAAGCAGGTTGTTGATGAGA
RUNX3 T2/3	**F** 5′-CGGTTCCACAGACAAGGAC<br>**R** 5′-CTAGAGCTCCAGCCGACTTC
RUNX3 E3	**F** 5′-GGATCTCAGCCATCACTTCC<br>**R** 5′-TGTGGAACCTGACAACAAGG
RUNX3 E4/5	**F** 5′-TGCCAAGTGAGAGTTCTGGA<br>**R** 5′-ATGTGAAGGCTGAACGAGGT
RUNX3 E6	**F** 5′-AGCTTCCGACCGTTGGTG<br>**R** 5′-AGCTTCCGACCGTTGGTG
RUNX3 T6	**F** 5′-TGACCTGACCTTCACATCAGA<br>**R** 5′-TCAGTGGTGCATAGGTGTCAG
RUNX1 E1	**F** 5′-GGTTCCCTGACAGCTGAACAT<br>**R** 5′-CCGCTCCCTTCTCGCATTTA
RUNX1 T1/2	**F** 5′-ACAAGCCTGCTCCTCTTCAC<br>**R** 5′-ACAAGAAGCATGACACCAACA
RUNX1 E2	**F** 5′-TGTAGGAAGTGGTATGGCAATG<br>**R** 5′-ATCTGACAAGCTCAGTGAGTGAA
RUNX1 E3	**F** 5′-ACTGGTCTGTGAAGCGGATAA<br>**R** 5′-AGCACTTCTTCCTCCGACAA
RUNX1 E4	**F** 5′-TGCCTGAGAACAGGTTGCTA<br>**R** 5′-GAGGACCTCAAGCTGAATCAA
RUNX1 T4/5	**F** 5′-GGCCTCATATTGGCACACTT<br>**R** 5′-CATAACTGTGGCCTCCACCT
RUNX1 E5	**F** 5′-CACTCCTTCGACTGAGCAGTTA<br>**R** 5′-CATGCTATCCACATGACATCACT
RUNX1 E6	**F** 5′-AGGTTCACCTCTCTGTCCTCTCT<br>**R** 5′-TGAGAATGCATGTGTGAGCA

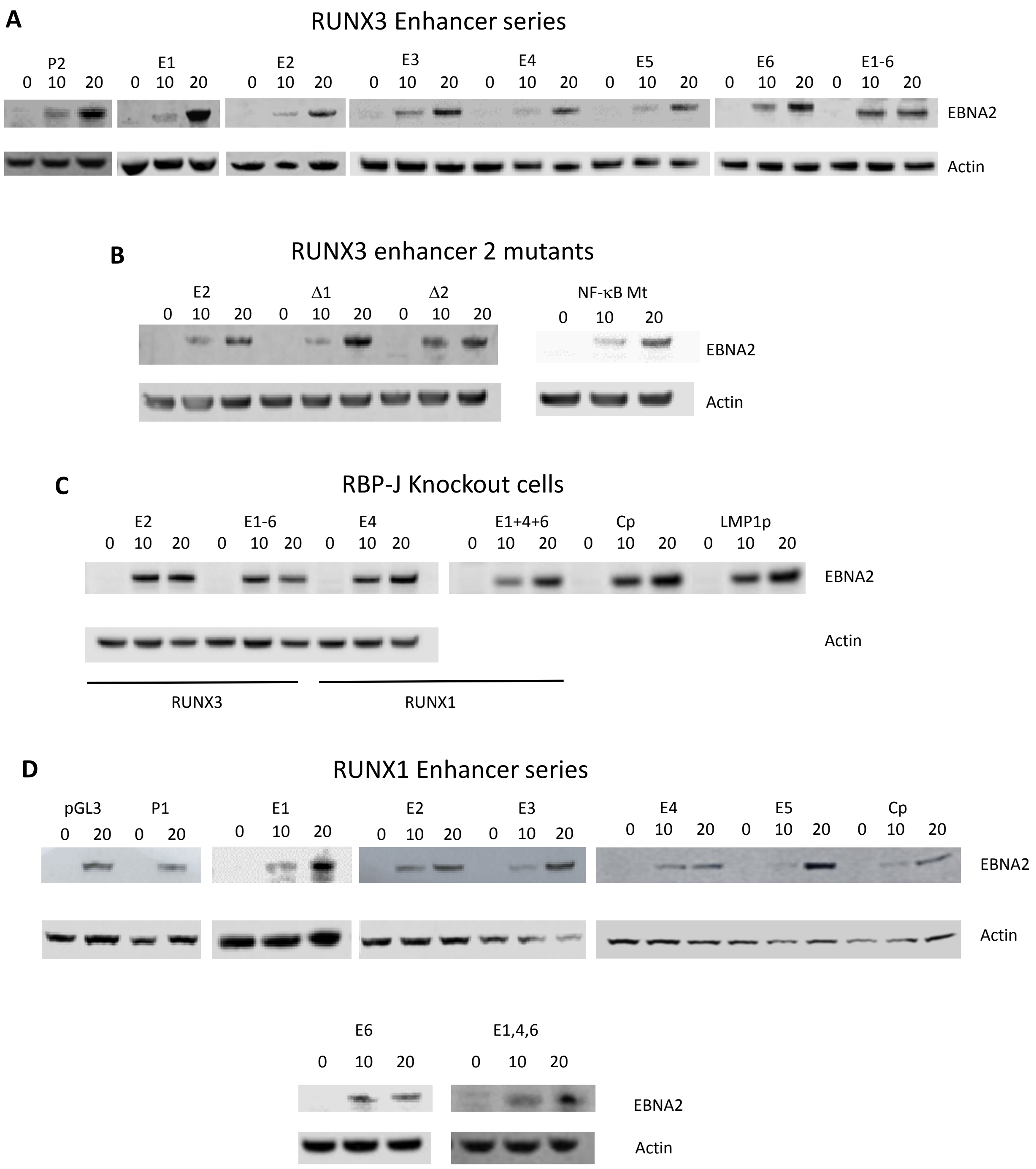

Supplementary Figure S1. Western blot analysis of representative luciferase assay samples. Parallel samples were processed for luciferase assays and Western blot analysis to detect EBNA2 protein expression. Actin levels serve as a loading control. (**A**) Analysis of cells transfected with *RUNX3* reporter constructs containing the P2 promoter alone (P2) or in the additional presence of each enhancer cloned upstream of the promoter either alone (E1, E2, E3, E4, E5, E6) or in combination (E1-6). (**B**) Analysis of cells transfected with wildtype *RUNX3* enhancer 2 and deletion or NF-κB enhancer 2 mutants. (**C**) Analysis of *RUNX1* and *RUNX3* reporter assay transfections in RBP-J knock-out cells. (**D**) Analysis of cells transfected with *RUNX1* reporter constructs containing P1 alone or in the additional presence of each enhancer cloned upstream of the promoter either alone (E1, E2, E3, E4, E5, E6) or E1,4, and 6 combined.

A

EBF1	1000
RUNX3	1000
PAX5	1000
USF1	1000
BATF	724
USF2	662
MEF2A	591
MAZ	410
EGR1	309
MEF2C	302
STAT5A	287
GABPA	270
YY1	270
RELA	249
ELK1	192
SP1	165
ELF1	159

B

PU.1	1000
RUNX3	1000
IRF4	544
POU2F2	296
EBF1	191

Supplementary Figure S2. Cellular TF binding to *RUNX* super-enhancers. ENCODE Factorbook cluster scores (out of 1000) for TF binding detected by ChIP-sequencing analysis in GM12878 cells. Only binding scores for TFs with a motif at their binding peak are shown. (A) Binding of TFs at *RUNX3* enhancer 2. (B) Binding of TFs at *RUNX1* enhancer 4.
