# Supplementary material for: RUNX super-enhancer control through the Notch pathway by Epstein-Barr virus transcription factors regulates B cell growth
Source: Nucleic Acids Res. 2016 Feb 15;44(10):4636–50. doi: 10.1093/nar/gkw085 (PMC4889917; doi:10.1093/nar/gkw085)
Supplement: SUPPLEMENTARY DATA [file supp_44_10_4636__index.html]

 RUNX super-enhancer control through the Notch pathway by Epstein-Barr virus transcription factors regulates B cell growth — RUNX super-enhancer control through the Notch pathway by Epstein-Barr virus transcription factors regulates B cell growth — SUPPLEMENTARY DATA 

# *RUNX* super-enhancer control through the Notch pathway by Epstein-Barr virus transcription factors regulates B cell growth

## SUPPLEMENTARY DATA

- SUPPLEMENTARY DATA
